# Molecular Characterization and Treatment Approaches for Pediatric *H3 K27*-Altered Diffuse Midline Glioma: Integrated Systematic Review of Individual Clinical Trial Participant Data

**DOI:** 10.3390/cancers15133478

**Published:** 2023-07-03

**Authors:** Sudarshawn Damodharan, Alexandra Abbott, Kaitlyn Kellar, Qianqian Zhao, Mahua Dey

**Affiliations:** 1 Department of Pediatrics, Division of Pediatric Hematology, Oncology and Bone Marrow Transplant, School of Medicine & Public Health, University of Wisconsin, Madison, WI 53792, USA; sdamodharan@uwhealth.org; 2 Department of Neurosurgery, School of Medicine & Public Health, University of Wisconsin, UW Carbone Cancer Center, Madison, WI 53792, USA; aaabbott2@wisc.edu (A.A.); kkellar@wisc.edu (K.K.); 3 Department of Biostatistics and Medical Informatics, School of Medicine and Public Health, University of Wisconsin, Madison, WI 53792, USA; qzhao36@wisc.edu

**Keywords:** pediatric, DMG, *H3 K27*-altered, clinical trials, overall survival, molecular

## Abstract

**Simple Summary:**

Diffuse midline glioma, *H3 K27*-altered are central nervous system tumors that primarily affect the pediatric population. Currently, there are no curative therapies, with several completed and on-going clinical trials exploring many different therapeutic options. In this systematic review, we utilized individual participant data from published clinical trials to assess the effect of treatment options, molecular makeup and clinical characteristics on overall survival. These findings are intended to provide guidance for future interventions along with indicate the need to continue to assess results from clinical trials as they become available at a larger scale.

**Abstract:**

Diffuse midline glioma (DMG), *H3 K27*-altered are highly aggressive, incurable central nervous system (CNS) tumors. The current standard palliative treatment is radiotherapy, with most children succumbing to the disease in less than one year from the time of diagnosis. Over the past decade, there have been significant advancements in our understanding of these heterogeneous tumors at the molecular level. As a result, most of the newer clinical trials offered utilize more targeted approaches with information derived from the tumor biopsy. In this systematic review, we used individual participant data from seven recent clinical trials published over the past five years that met our inclusion and exclusion criteria to analyze factors that influence overall survival (OS). We found that the most prominent genetic alterations H3.3 (*H3F3A*) and *TP53* were associated with worse OS and that ACVR had a protective effect. In addition, re-irradiation was the only statistically significant treatment modality that showed any survival benefit. Our findings highlight some important characteristics of DMG, *H3 K27*-altered and their effects on OS along with the importance of continuing to review clinical trial data to improve our therapies for these fatal tumors.

## 1. Introduction

Diffuse midline glioma (DMG), *H3 K27*-altered are devastating central nervous system (CNS) tumors that affect both children and adults. The most common locations of these tumors are within the pons, thalamus or spinal cord of the CNS. These tumors are designated as World Health Organization (WHO) grade 4 regardless of their histology and anatomical location. The characteristic *H3 K27M* mutation that is the hallmark of these tumors comes in two primary genotypes with mutations in the *HIST1H3B/C* (H3.1) and *H3F3A* (H3.3) genes. 

Our understanding of the pathogenesis and underlying molecular makeup of these tumors has improved over the past decade, yet there remains no cure. Conventional photon radiation therapy remains the standard treatment approach, with numerous clinical trials exploring more effective therapies. Over recent years, the clinical standard of DMG management has evolved to include regular biopsy and histological confirmation. In addition, with the increased use of next generation sequencing (NGS), numerous clinical trials have looked to assess the feasibility and efficacy of various targeted agents based on the molecular makeup of each individual patient’s tumors.

It is well known that those DMGs with the *H3 K27M* mutation have poorer outcomes as well as halted overall survival (OS) compared the unmutated type [1,2,3]. With newer therapies being utilized in clinical trials, we looked at more recently published literature to assess the efficacy of various treatments for pediatric patients with DMG, *H3 K27*-altered. Our primary objective was to investigate the impact of various treatment modalities and molecular alterations on OS. Our secondary analysis involved investigating other clinical factors, such as age, sex and tumor location, on DMG’s incidence as well as prognostic significance. This systematic analysis was performed by integrating the individual participant data (IPD) of recently published clinical trials that meet our inclusion/exclusion criteria. As newer therapies emerge, it will be important to continue to assess the response of these tumors in the context of their molecular makeup, given the significant heterogeneity of these tumors. 

## 2. Materials and Methods

### 2.1. Study Selection Criteria

This systematic analysis was registered via the National Institute for Health and Care Research (NIHR) via identification number 432005. A search strategy was developed utilizing the Populations, Intervention, Comparison, Outcome, Study Type (PICOS) question format. In pediatric patients with diffuse midline glioma, H3 K27-altered (Population) who received targeted therapy as part of a clinical trial (Intervention and Study Type), the effects on overall survival based on the therapeutic modality along with the known molecular alterations, anatomic location of the tumor, sex and age of the patient (Comparison and Outcome) were determined. Though any H3 K27 mutation could be included within our study, all of the patients had the presence of the H3 K27M missense mutation.

**Figure 1:**
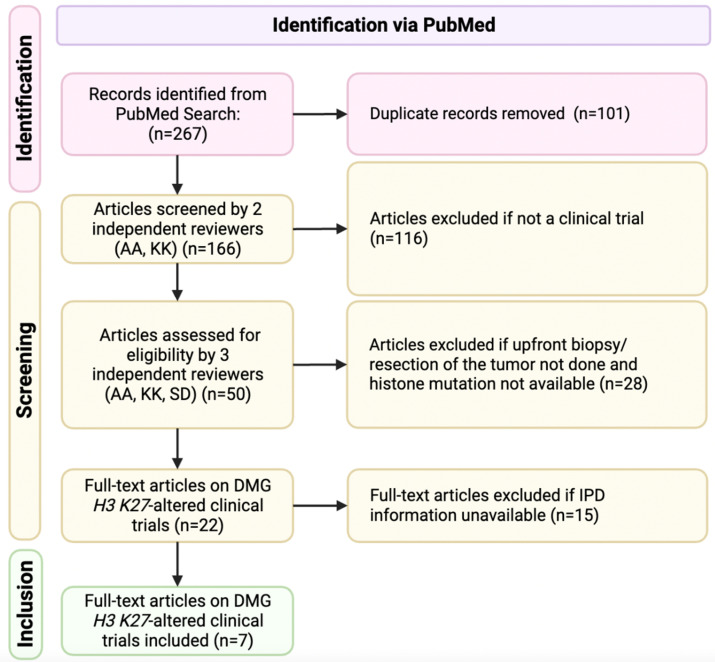
Flow diagram of systematic review according to PRISMA guidelines.

A literature search in PUBMED was performed by three independent reviewers according to the Preferred Reporting Items for Systematic Reviews and Meta-Analysis (PRISMA) (Figure 1) guidelines. Our search included clinical trials, published from 1 January 2018 to 2 March 2023. The terms used for the literature search included “diffuse midline glioma” OR “diffuse intrinsic pontine glioma” OR “DMG” OR “DIPG” AND “targeted therapy” OR “clinical trial” OR “molecular”. These terms were specifically used in batch searches across both databases. All the articles resulting from our searches were analyzed for our inclusion and exclusion criteria and only articles that satisfied all our criteria were selected for the final analysis (Table 1).

### 2.2. Quality Evaluation of Clinical Trials

Publications included in this study were evaluated according to the Newcastle–Ottawa Scale (NOS) to assess for quality of the included study. This assessment tool has three domains: selection (maximum 4 stars), comparability (maximum 2 stars) and outcome (maximum 3 stars) [4]. Studies that were awarded fewer than 6 stars were considered low quality while those with at least 6 stars were regarded as moderate- to high-quality studies (Table 2). 

### 2.3. Data Extraction

All IPD were extracted from the main manuscript and supplementary text, tables and figures of the included articles. In total, 108 patients were identified from 7 published reports from published clinical trials (Table 3). Our primary outcome of interest was patients’ overall survival treated with the various therapies compared to one another. Secondary outcomes included assessing tumor anatomical location, sex, age and their prognostic impact. Two independent investigators (A.A. and K.K.) extracted the data, and confirmation of extracted data was also conducted by a third independent reviewer (S.D.). Cases that did not undergo an upfront biopsy or did not have the *H3 K27M* mutation were subsequently removed. 

### 2.4. Statistical Analysis

The primary outcome from our study was impact of treatment modalities and molecular alterations on the OS of patients with DMG, H3 K27-altered. Additional factors were also assessed, including anatomic location of the tumor, sex and age of the patient, to determine their effects on OS along with co-incidence of one another. Statistical analysis was conducted using SAS software (SAS Institute Inc., Cary, NC, USA), version 9.4. All reported *p*-values are two-sided and a *p* < 0.05 was used to define statistical significance. Descriptive statistics such as count and frequency were generated for demographic variables. ANOVA and T-test were used to test difference of overall survival between different demographics, anatomical location, gene mutations and treatments. Fisher’s exact was used to test the association between gene mutations, treatments and anatomical location and sex.

## 3. Results

### 3.1. Study Characteristics

Our initial PubMed search resulted in 267 publications. The clinical trial article type was selected, which eliminated 116 articles. Due to duplicity, an additional 101 articles were eliminated. Forty-three articles were eliminated after the title/abstract were screened based upon our inclusion and exclusion criteria or if no IPD were available. In total, seven studies were selected for full-text review. By reading the full text of these papers, we integrated the IPD from all seven studies for the final analysis [5,6,7,8,9,10,11]. We identified a total of 108 pediatric cases with the available pertinent clinical data. Of note, 34 patients did not have NGS results available, either due to an inadequate sample or sampling not being performed. All included studies were rated as moderate- to high-quality based upon the NOS assessment tool. Table 2 presents the studies included in this analysis. 

All patients included in our analysis underwent biopsy/partial resection of the initial tumor without any further surgeries conducted. The confirmation of the *H3 K27M* mutation was performed via IHC along with sequencing for all patients to delineate the H3.3 or H3.1 sub-type. 

### 3.2. Survival Differences

We found overall survival differences stratified against molecular alterations and therapeutic interventions. Additional survival differences were seen in the age distribution of pediatric patients with DMG, H3 K27-altered. Patients aged <5 years had better OS (20.50 months) compared to patients aged >10 years (14.99 months) (*p* < 0.05). Tumors carrying the H3.3 (*H3F3A*) genotype had a worse OS compared to those with the H3.1 (*HIST1H3B*/*C*) (OS 14.25 months vs. 19.62 months; *p* < 0.001). Additionally, the tumors expressing the TP53 alteration also had a worse OS (13.71 months vs. 21.41 months; *p* < 0.0001), whereas those that expressed *ACVR* had an improved OS when compared against all the gene alterations that were assessed for (20.85 months vs. 14.89 months; *p* = 0.0081). Regarding therapeutic interventions, the only statistically significant treatment was tumor re-irradiation (18.62 months vs. 14.58 months; *p* = 0.0047). The use of any personalized treatment based upon the patients’ NGS alterations also showed a marginal benefit, though not statistically significant. The patients’ sex or anatomic location of the tumor had no effect on OS in our analysis (Table 4). 

### 3.3. Genetic Alterations

**Figure 2:**
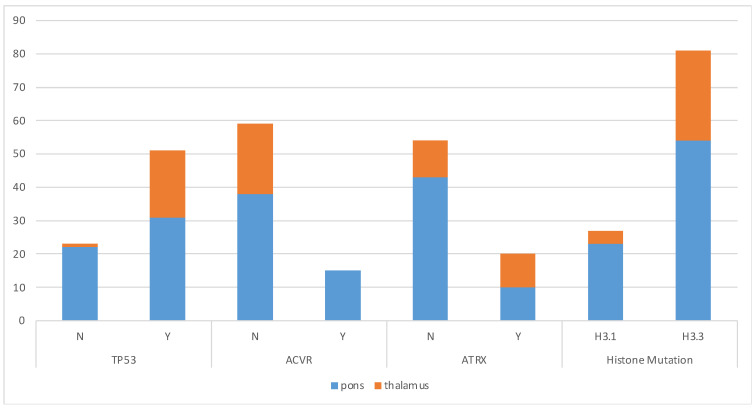
Significant molecular alterations by anatomical location.

Differences were observed in the type of genetic alterations seen when compared to the anatomic location of the tumor along with the sex of the patient. *TP53* was identified frequently in both pontine and thalamic tumors (pons present in 58% vs. absent in 42% and thalamus present in 95% vs. absent in 5%; *p* 0.0018); however, it was proportionally more significant within thalamic tumors without any significant differences in sex. *ACVR* was solely identified in pontine tumors without any thalamic tumors showing expression for this and was more prevalent in female patients (31% vs. 9%; *p* = 0.0218). Thalamic tumors had a higher prevalence of *ATRX* mutations in comparison to tumors within the pons (48% vs. 10%; *p* = 0.0194). The H3.3 (*H3F3A*) alteration was more prevalent in both the pons and thalamus and in both male and female patients compared to H3.1 (*HIST1H3B*/*C*), without any statistical significance noted (Figure 2 and Table 5).

### 3.4. Treatment and Tumor Location

The type of treatments utilized when looking at the anatomical location of the tumor did show significant differences in the IPD analysis from the clinical trials. The use of a personalized treatment approach, based on an individual patient’s NGS sequencing, was more commonly utilized in pontine tumors compared to those within the thalamus (98% vs. 2%; *p* < 0.0001). This was also observed with patients who received a combination therapy of nimotuzumab and vinorelbine, where the combination therapy was only used for pontine tumors. On the other hand, the use of temozolomide was seen within both anatomic locations but was more prevalent in thalamic tumors. Immunotherapeutic approaches were also used for both anatomic locations but were more often used in pontine tumors (82% vs. 18%; *p* = 0.0015). Lastly, both re-irradiation and other chemotherapeutic therapies were used in patients with tumors in both locations but were more commonly seen in pontine tumors (Table 6). 

## 4. Discussion

DMG, *H3 K27*-altered are fatal tumors located in midline CNS structures, primarily in the brainstem and thalamus but they can also be present within the spinal cord. The overall survival for pediatric patients is less than one year, generally ranging from 8–12 months after initial diagnosis [12,13]. Over the last decade, our understanding of the molecular makeup and biology of these tumors has expanded significantly. Now, biopsy is routinely conducted for tumors present within the pontine region (diffuse intrinsic pontine glioma or DIPG), and NGS testing is standard for the characteristic histone mutations associated with tumors present in other midline locations, such as the thalamus. With the advent of NGS, a more personalized approach to treatment has been the center of numerous clinical trials for these heterogeneous tumors, yet it is not certain how beneficial these treatments have been compared to the historical norm at a larger scale. 

In this study, we integrated IPD from 108 biopsied or resected DMG, *H3 K27*-altered patients that participated in a clinical trial. Our study represents the first meta-analysis assessing the integrated response of DMG, *H3 K27*-altered patients enrolled in clinical trials published within the last 5 years. All patients in our study had confirmed presence of the *H3 K27M* mutation and the specific variant confirmed via sequencing. Most of the patients had NGS run on their tumor tissue, though many had inadequate or insufficient tissue for this to be assessed (34 in total). We primarily looked at the use of various treatment options and molecular alterations and their effect on OS in our patient cohort. We also assessed further available clinical data including age, sex and anatomical location for their effect on OS along with the incidence of molecular alterations and use of therapeutic strategies. 

Our analysis showed that molecular characteristics of the tumor have significant impacts on patients’ overall outcome, with patients who had the H3.3 (*H3F3A*) genotype and *TP53* mutation carrying a worse OS. Conversely, patients with an *ACVR* mutation had an improved OS compared to the rest. It has been well described that patients who carry the H3.3 genotype of DMG, *H3 K27*-altered have a halted OS and more protracted course [14,15,16]. The exact reasoning for this is not known, but irrespective of anatomical location has been found to be true [17,18]. Similarly, the *TP53* mutation has also portrayed a poorer prognosis along with a high occurrence in patients with the H3.3 genotype [19,20]. It has been thought that *TP53* may play a role in radio resistance in these tumors, leading to a halted OS compared to those without it [21,22]. The prevalence of *TP53* was seen in thalamic tumors at a higher percentage compared to those in the pons in our study. Within the literature, the incidence of *TP53* has not been well established with the anatomical location of the tumor but has been shown to be prevalent within all midline locations [1,23]. The *ACVR* mutation showed a protective effect on OS in our analysis. As a single gene alteration level, this has not been looked at in this regard, and further analysis is warranted to better understand and validate it given the fatality of the tumor. The prevalence of *ACVR* was seen only in the pontine location in our study, which is true considering what has also been described in similar studies of DMG, *H3 K27*-altered [18,24,25]. Sex differences have not been well described for this tumor, and aside from a higher incidence of *ACVR* in female patients compared to male in our analysis, no other differences were seen. 

Previous studies have refuted the notion that anatomical location had an impact on the OS of DMG, *H3 K27*-altered in pediatric patients [18,26]. This was also the case in our study. However, more recent literature suggests this to not be true, with tumors within the brainstem and pons region having a decreased OS compared to tumors located in other midline areas [18,27,28]. The literature suggests that older patients typically have a more prolonged OS [29,30,31]. This was different from what was seen in our analysis, with patients <5yo having the longest OS and patients between 5–10yo having the worst OS. This is limited by the number of patients accounting for the <5yo and >10yo groups in our study, which limits the clinical significance to this. Therapeutically, only irradiation was found to have a significant benefit on OS. The literature also corroborates this, but the toxicity and poor quality of life associated with repeated radiation and its marginal improvement on OS has made this treatment modality unappealing [32,33]. The use of personalized therapeutic strategies showed a marginal benefit in our analysis, and this is likely the approach that will be needed at a combination level to combat these highly heterogeneous tumors. The use of most therapeutic interventions assessed in our study were seen to a higher degree in pontine tumors compared to thalamic. This is confounded by the increased number of pontine tumors in our study, but also proportionally seen within the literature with the pons being the primary location of DMG, *H3 K27*-altered in the pediatric population. 

Conventional photon radiation therapy remains the standard palliative treatment offered to patients, but currently there are 11 interventional clinical trials enrolling with the majority utilizing a targeted therapy or combination approach (Table 7). As shown in previous studies, the use of oral chemotherapy agents such as temozolomide along with immunotherapeutic options such as bevacizumab did not seem to make a difference in OS while also adding more adverse effects for patients [34,35,36,37]. As shown from our study, the use of these treatments showed similar results, highlighting the need for further improvement for a sustained response while minimizing toxicity. One avenue that has shown promise is the use of other immunotherapeutic options such as CAR T cell therapy, with further research needed to explore this as a sustainable treatment [38,39,40,41]. Continued research looking into novel therapeutic targets along with more combinatorial therapeutic approaches is on-going. 

The results of our study add to the existing current knowledge known for pediatric patients with DMG, *H3 K27*-altered. By utilizing patients who had been enrolled in clinical trials, we were able to assess the response of various therapeutic options and genetic alterations on OS. Additional factors including age, sex and anatomical location of tumor were also analyzed for their impact on OS. The limitations of our study include the overall small sample size and higher proportion of pontine tumors and patients aged 5–10yo. As shown in Table 3, many patients from the utilized studies were also excluded from analysis as they did not have a biopsy performed, which contributed to the heterogeneity of our cohort. As more results from on-going and completed clinical trials come about with the use of targeted, immunotherapeutic and personalized approaches using molecular characterization, it will be prudent to re-analyze this topic for further differences or notable changes at a larger scale. 

## 5. Conclusions

Our study demonstrated that the molecular characteristics of pediatric DMG, *H3 K27*-altered influences on overall outcome, though the tumor still remains universally fatal. Additional analysis showed that the *ACVR* mutation was associated with a prolonged OS compared to patients without this and that those with an H3.3 genotype and *TP53* mutation were associated with worse survival. No sex differences were observed in our study regarding OS. Personalized therapeutic interventions showed a marginal benefit in our cohort, with more investigation warranted. With the increased use of targeted and combination therapies currently and in upcoming clinical trials, further research and evaluation of clinical trial data are needed to continue to help advance our understanding and management of these fatal tumors. 

## Figures and Tables

**Table 1:** Inclusion and exclusion criteria.

Inclusion	Exclusion
(1) patients <18yo	(1) patients >18yo
(2) clinical trial publications	(2) non-clinical trial publications
(3) published in English	(3) published in languages different than English
(4) published within the last 5 years (2018 and onwards)	(4) published prior to 2018
(5) upfront tumor biopsy/resection done	(5) upfront tumor biopsy/resection not done
(6) histone mutation status available	(6) histone mutation status not available
(7) IPD available	(7) IPD unavailable

**Table 2:** Included studies and quality assessment.

Authors & Year	Country	NOS (No. of Stars)		
		Selection	Comparability	Outcome
Del Baldo et al., 2022 [5]	Italy	4	0	3
DeWire et al., 2020 [6]	USA	4	0	3
El-Khouly et al., 2021 [7]	Netherlands	4	0	2
Pérez-Larraya et al., 2022 [8]	Spain	4	0	2
Gojo et al., 2020 [9]	Austria	4	0	3
Mueller et al., 2019 [10]	USA	4	0	3
Rodriguez et al., 2022 [11]	Italy	4	0	3

**Table 3:** Characteristics of included studies.

Articles	Country	Trial Overview/Intervention	Total Number Participants	Participants Included in Analysis
Del Baldo et al., 2022 [5]	Italy	Targeted therapies combined with standard of care for pediatric DMG.	25	25
DeWire et al., 2020 [6]	USA	Addition of ribociclib following standard-of-care radiotherapy for DMG.	10	10
El-Khouly et al., 2021 [7]	Netherlands	Combined treatment with bevacizumab, irinotecan and erlotinib for DMG.	9	4
Gojo et al., 2020 [9]	Austria	Feasibility and outcomes of including personalized treatment via molecular tumor analysis with focal irradiation and backbone therapy for DMG.	18	17
Mueller et al., 2019 [10]	USA	Evaluated whether whole-exome sequencing and RNA sequencing of paired normal and tumor tissues could be incorporated into personalized treatment of DMG.	17	13
Pérez-Larraya et al., 2022 [8]	Spain	Dose-escalation study of DNX-2401, an oncolytic adenovirus, followed by radiotherapy for DMG.	12	10
Rodriguez et al., 2022 [11]	Italy	Bevacizumab in combination with temozolomide and radiotherapy in pediatric patients with high-grade glioma.	121	29

**Table 4:** Characteristics, molecular alterations and treatments used in pediatric DMG, H3 K27-altered.

Parameters		Number of Patients	Overall Survival		*p*-Value
			Mean	STD	
Age	<5	16	20.50	9.72	0.0163 *
	5–10	52	14.54	6.07	
	10+	40	14.99	7.69	
Sex	Female	57	15.61	6.84	0.9845
	Male	51	15.58	8.30	
Anatomical location	pons	77	16.30	7.41	0.1229
	thalamus	31	13.83	7.63	
Histone Mutation	H3.1 (HIST1H3B/C)	27	19.62	7.67	0.0011 *
	H3.3 (H3F3A)	18	14.25	7.02	
TP53	N	23	21.41	8.38	<0.0001 *
	Y	51	13.71	6.40	
ACVR	N	59	14.89	7.16	0.0081 *
	Y	15	20.85	9.03	
ATRX	N	54	16.26	7.94	0.7698
	Y	20	15.66	7.91	
PIK3CA	N	63	15.59	7.29	0.1824
	Y	11	19.04	10.63	
mTOR	N	63	15.52	7.95	0.1275
	Y	11	19.45	6.84	
BRAF	N	71	15.87	7.72	0.2311
	Y	3	21.47	11.59	
PDGFRA	N	60	16.87	8.22	0.0838
	Y	14	12.82	5.27	
FGFR3	N	70	16.19	8.04	0.6855
	Y	4	14.53	4.56	
Personalized treatment approach	N	59	14.33	6.57	0.0548
	Y	49	17.12	8.35	
Nimotuzumab/vinorelbine	N	74	14.86	7.36	0.1368
	Y	34	17.19	7.74	
Temozolomide	N	49	14.68	7.76	0.2539
	Y	59	16.35	7.30	
Bevacizumab	N	61	14.89	8.08	0.2683
	Y	47	16.51	6.71	
Panobinostat	N	101	15.65	7.64	0.7551
	Y	7	14.73	6.08	
Immunotherapy	N	37	14.17	7.77	0.1576
	Y	71	16.33	7.34	
Other chemotherapy	N	66	14.88	6.90	0.2163
	Y	42	16.72	8.38	
Re-irradiation	N	81	14.58	7.86	0.0047 *
	Y	27	18.62	5.52	
Radiation	N	9	17.69	8.80	0.3843
	Y	99	15.40	7.42	

Y = yes; N = no; * = statistical significance; other chemotherapy = systemic chemotherapy regimens not otherwise assessed as above.

**Table 5:** Gene alteration by tumor location and sex.

Gene		Anatomical Location			Sex		
		Pons	Thalamus	*p*-Value	Female	Male	*p*-Value
TP53	N	22 (42%)	1 (5%)	0.0018 *	14 (36%)	9 (26%)	0.4519
	Y	31 (58%)	20 (95%)		25 (64%)	26 (74%)	
ACVR	N	38 (72%)	21 (100%)	0.004 *	27 (69%)	32 (91%)	0.0218 *
	Y	15 (28%)	0 (0%)		12 (31%)	3 (9%)	
ATRX	N	43 (81%)	11 (52%)	0.0194 *	28 (72%)	26 (74%)	1.0000
	Y	10 (19%)	10 (48%)		11 (28%)	9 (26%)	
PIK3CA	N	46 (87%)	17 (81%)	0.4953	33 (85%)	30 (86%)	1.0000
	Y	7 (13%)	4 (19%)		6 (15%)	5 (14%)	
mTOR	N	43 (81%)	20 (95%)	0.1628	31 (79%)	32 (91%)	0.1980
	Y	10 (19%)	1 (5%)		8 (21%)	3 (9%)	
BRAF	N	51 (96%)	20 (95%)	1.0000	39 (100%)	32 (91%)	0.1010
	Y	2 (4%)	1 (5%)		0 (0%)	3 (9%)	
PDGFRA	N	43 (81%)	17 (81%)	1.0000	31 (79%)	29 (83%)	0.7732
	Y	10 (19%)	4 (19%)		8 (21%)	6 (17%)	
FGFR3	N	51 (96%)	19 (90%)	0.3180	37 (95%)	33 (94%)	1.0000
	Y	2 (4%)	2 (10%)		2 (5%)	2 (6%)	
Histone Mutation	H3.1 (HIST1H3B/C)	23 (30%)	4 (13%)	0.0861	15 (26%)	12 (24%)	0.8254
	H3.3 (H3F3A)	54 (70%)	27 (87%)		42 (74%)	39 (76%)	

Y = yes; N = no; * = statistical significance.

**Table 6:** Treatment use by tumor anatomical location.

Treatment		Anatomical Location		
		Pons	Thalamus	*p*-Value
Personalized treatment approach	N	29 (49%)	30 (51%)	<0.0001 *
	Y	48 (98%)	1 (2%)	
Nimotuzumab/vinorelbine	N	43 (58%)	31 (42%)	<0.0001 *
	Y	34 (100%)	0 (0%)	
Temozolomide	N	48 (98%)	1 (2%)	<0.0001 *
	Y	29 (49%)	30 (51%)	
Bevacizumab	N	43 (70%)	18 (30%)	1.0000
	Y	34 (72%)	13 (28%)	
Panobinostat	N	70 (69%)	31 (31%)	0.1887
	Y	7 (100%)	0 (0%)	
Immunotherapy	N	19 (51%)	18 (49%)	0.0015 *
	Y	58 (82%)	13 (18%)	
Other chemotherapy	N	36 (55%)	30 (45%)	<0.0001 *
	Y	41 (98%)	1 (2%)	
Re-irradiation	N	51 (63%)	30 (37%)	0.0005 *
	Y	26 (96%)	1 (4%)	
Radiation	N	8 (89%)	1 (11%)	0.4416
	Y	69 (70%)	30 (30%)	

Y = yes; N = no; * = statistical significance.

**Table 7:** Available clinical trials for pediatric DMG, H3 K27-altered.

Study Title	NCT Number	Therapeutic Intervention	Country
Stereotactic Biopsy Split-Course Radiation Therapy in Diffuse Midline Glioma, SPORT-DMG Study	NCT05077735	Radiation: hypofractionated radiation therapy	USA
A Study of BXQ-350 in Children with Newly Diagnosed Diffuse Intrinsic Pontine Glioma (DIPG) or Diffuse Midline Glioma (DMG)	NCT04771897	Drug: BXQ-350	USA
FUS Etoposide for DMG—A Feasibility Study	NCT05762419	Drug: etoposide and device: focused ultrasound with neuro-navigator-controlled sonication	USA
rHSC-DIPGVax Plus Checkpoint Blockade for the Treatment of Newly Diagnosed DIPG and DMG	NCT04943848	Biological: rHSC-DIPGVax; drug: balstilimab; and drug: zalifrelimab	USA
Biological Medicine for Diffuse Intrinsic Pontine Glioma (DIPG) Eradication 2.0	NCT05476939	Drug: everolimus; drug: ONC201; and radiation: radiotherapy	France
Combination Therapy for the Treatment of Diffuse Midline Gliomas	NCT05009992	Drug: ONC201; radiation: radiation therapy; and drug: paxalisib	USA
A Study of the Drug Selinexor with Radiation Therapy in Patients with Newly-Diagnosed Diffuse Intrinsic Pontine (DIPG) Glioma and High-Grade Glioma (HGG)	NCT05099003	Radiation: radiation therapy and drug: selinexor	USA
Loc3CAR: Locoregional Delivery of B7-H3-CAR T Cells for Pediatric Patients with Primary CNS Tumors	NCT05835687	Drug: B7-H3-CAR T cells	USA
Phase I Study of Oral ONC206 in Recurrent and Rare Primary Central Nervous System Neoplasms	NCT04541082	Drug: ONC206	USA
Oral AMXT 1501 Dicaprate in Combination with IV DFMO	NCT05500508	Drug: AMXT1501 and drug: DFMO	USA
ONC206 for Treatment of Newly Diagnosed, or Recurrent Diffuse Midline Gliomas, and Other Recurrent Malignant CNS Tumors (PNOC 023)	NCT04732065	Drug: ONC206 and radiation: radiation therapy	USA and Switzerland

Clinical trials found on www.clinicaltrials.gov as of 25 May 2023.
